# Murine Precision-Cut Kidney Slices as an *ex vivo* Model to Evaluate the Role of Transforming Growth Factor-β1 Signaling in the Onset of Renal Fibrosis

**DOI:** 10.3389/fphys.2017.01026

**Published:** 2017-12-12

**Authors:** Elisabeth G. D. Stribos, Marc A. Seelen, Harry van Goor, Peter Olinga, Henricus A. M. Mutsaers

**Affiliations:** ^1^Department of Pharmaceutical Technology and Biopharmacy, Groningen Research Institute of Pharmacy, University of Groningen, Groningen, Netherlands; ^2^Division of Nephrology, Department of Internal Medicine, University of Groningen, University Medical Center Groningen, Groningen, Netherlands; ^3^Division of Pathology, Department of Pathology and Medical Biology, University of Groningen, University Medical Center Groningen, Groningen, Netherlands

**Keywords:** precision-cut kidney slices, *ex vivo* model, renal fibrosis, transforming-growth factor β, antifibrotic therapies, chronic kidney diseases

## Abstract

Renal fibrosis is characterized by progressive accumulation of extracellular matrix (ECM) proteins, resulting in loss of organ function and eventually requiring renal replacement therapy. Unfortunately, no efficacious treatment options are available to halt renal fibrosis and translational models to test pharmacological agents are not always representative. Here, we evaluated murine precision-cut kidney slices (mPCKS) as a promising *ex vivo* model of renal fibrosis in which pathophysiology as well as therapeutics can be studied. Unique to this model is the use of rodent as well as human renal tissue, further closing the gap between animal models and clinical trials. Kidneys from C57BL/6 mice were used to prepare mPCKS and slices were incubated up to 96h. Viability, morphology, gene expression of fibrosis markers (*Col1a1, Acta2, Serpinh1, Fn1*, and *Pai-1*), inflammatory markers (*Il1b, Il6, Cxcl1*), and protein expression (collagen type 1, α-smooth muscle actin, HSP47) were determined. Furthermore, to understand the role of the transforming-growth factor β (TGF-β) pathway in mPCKS, slices were incubated with a TGF-β receptor inhibitor (LY2109761) for 48 h. Firstly, viability and morphology revealed an optimal incubation period of 48 h. Secondly, we demonstrated an early inflammatory response in mPCKS, which was accompanied by subsequent spontaneous fibrogenesis. Finally, LY2109761 showed great antifibrotic capacity in mPCKS by decreasing fibrosis markers on mRNA level as well as by reducing HSP47 protein expression. To conclude, we here present an *ex vivo* model of renal fibrosis, which can be used to further unravel the mechanisms of renal fibrogenesis and to screen antifibrotic therapy efficacy.

## Introduction

Renal fibrosis is an integral part of the pathophysiological mechanism underlying the development and progression of chronic kidney disease (CKD), and is regarded as the most damaging process responsible for renal function decline (Schanstra et al., 2015). CKD affects about 10% of the population and substantially impacts health care budgets. Moreover, the global incidence of CKD is continuously rising (Couser et al., 2011; Jha et al., 2013). CKD is irreversible and can progress to end-stage renal disease, ESRD, (estimated glomerular filtration rate < 15 ml/min/1.78 m^2^) whereby renal replacement therapy such as dialysis or renal transplantation is needed.

Renal fibrosis is the consequence of an imbalanced extracellular matrix (ECM) turnover caused by continuous noxious stimuli such as trauma, infection, ischemia, or a systemic disease (Declèves and Sharma, 2014; Mutsaers et al., 2015). A multitude of complex pathways are implicated in the pathogenesis of renal fibrosis of which the transforming growth factor (TGF)-β pathway is regarded as the master regulator (Eddy and Fogo, 2006; Meng et al., 2016). Binding of TGF-β to a serine-threonine kinase type II receptor results in the recruitment and phosphorylation of a type I receptor, which in turn phosphorylates SMADs thereby initiating a host of signaling cascades (Massagué, 2012; Rockey et al., 2015). TGF-β is synthesized and secreted by inflammatory cells and a variety of effector cells. Activation of the pathway results in the formation and deposition of ECM proteins, mainly by activated interstitial (myo)fibroblasts (Rockey et al., 2015).

A myriad of experimental treatment modalities for renal fibrosis have been developed in hopes of retarding or even reversing fibrogenesis (Lee et al., 2015). Although several studies have been successful at the pre-clinical level, only limited advances have been made in the translation of these findings to the level of patient treatment. One of the limiting factors during the drug development process is the translation from *in vitro/vivo* models to the clinic. While *in vitro* models lack cellular heterogeneity, animal experiments do not fully reflect the human situation and results differ per strain (Inoue et al., 2015). To achieve a greater understanding of renal fibrosis and thus accelerate the discovery of effective therapeutic targets, there is an urgent need for adequate translational models. To this end, precision-cut kidney slices (PCKS) have been developed as disease model using either murine or human renal tissue (Poosti et al., 2015; Stribos et al., 2016a,b). Murine PCKS (mPCKS) are a promising model of renal fibrosis pathophysiology as well as an easy drug-discovery tool having several advantages compared to human PCKS such as tissue availability, use of knockout models and identical genetic background. Moreover, the use of PCKS contributes to the reduction, refinement and replacement (“3Rs” principles) of animal studies. In this study, we extensively characterized the phenotype of mPCKS during extended culture (up to 96 h) and studied the mechanisms behind fibrogenesis in PCKS.

## Materials and methods

### Chemicals

LY2109761, a TGF-β receptor type I/II (TβRI/II) dual inhibitor, was purchased from Selleck Chemicals, Munich, Germany. Stock solutions were prepared in DMSO and stored at −20°C. During the experiments, stocks (5 mM) were diluted in culture medium with a final solvent concentration of ≤0.1%.

### Animals

Tissue from male C57BL/6 mice (University Medical Center Groningen, Groningen, the Netherlands) aged 8–15 weeks was used for the experiments (*n* = 3 for long-term incubation studies and *n* = 4–5 for studies with LY2109761). Animals were housed under controlled conditions with a 12 h light/dark cycle and free access to water and food (Harlan chow no. 2018, Horst, the Netherlands). Organs were harvested via a terminal procedure performed under isoflurane/O_2_ anesthesia. Kidneys were stored in ice-cold University of Wisconsin (UW) organ preservation solution until use. The animal experiments were evaluated and approved by the Animal Ethics Committee of the University of Groningen (DEC 6416AA-001).

### Murine precision-cut kidney slices

**Figure 1:**
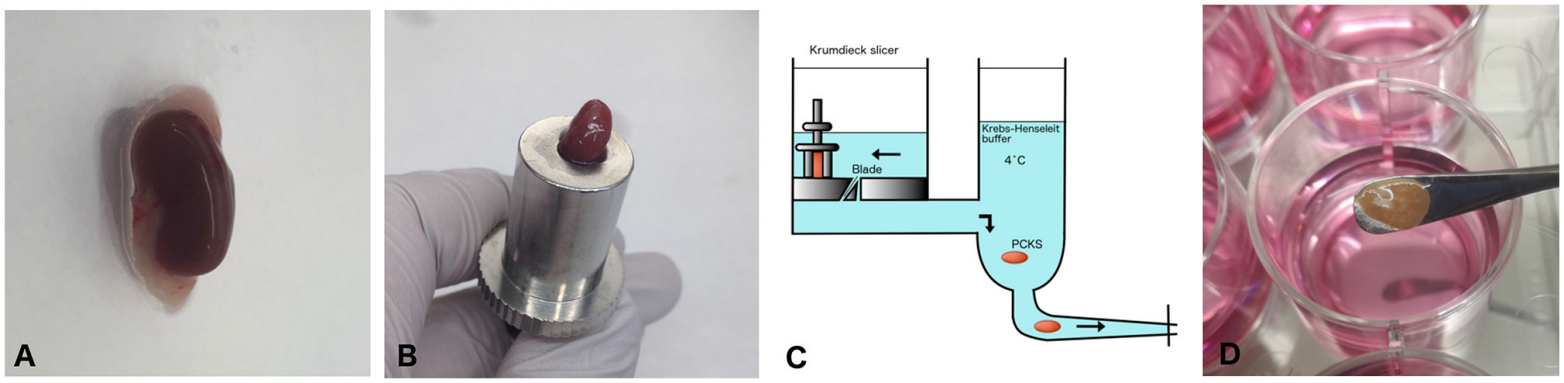
Workflow preparation of mPCKS. **(A)** Murine kidneys are prepared by removing the adipose tissue. **(B)** The entire kidney is placed in the core holder. **(C)** Murine PCKS (250–300 μm in thickness) are prepared with the Krumdieck slicer and subsequently kept in ice-cold University of Wisconsin (UW) solution. **(D)** Slices are incubated in optimized medium at 37°C and 80% O_2_, 5% CO_2_ in a gently shaking incubator. Adapted from Stribos et al. (2016b); used with permission from Elsevier. PCKS, precision-cut kidney slices.

Murine PCKS were prepared as described in detail by Poosti et al. (2015). In short, murine kidneys were prepared by removing adipose tissue and slices were made in ice-cold Krebs-Henseleit buffer supplemented with 25 mM D-glucose (Merck, Darmstadt, Germany), 25 mM NaHCO_3_ (Merck), 10 mM HEPES (MP Biomedicals, Aurora, OH, USA), and saturated with carbogen (95% O_2_, 5% CO_2_) using a Krumdieck tissue slicer (Figure 1). The obtained slices were 4.5 mm in diameter and had a wet weight of 4–6 mg, corresponding to an estimated thickness of 250–300 μm. Slices were incubated, up to 96 h, in Williams' Medium E with GlutaMAX (Life Technologies, Carlsbad) supplemented with 10 μg/mL ciprofloxacin and 2.7 g/L D-(+)-Glucose solution (Sigma-Aldrich, Saint Louis) at 37°C in an 80% O_2_ and 5% CO_2_ atmosphere while gently shaken. Medium was refreshed every 24 h. Murine PCKS were also treated for 48 h with 2.5 μM LY2109761, a TGF-β receptor inhibitor. After incubation, mPCKS were snap-frozen in liquid nitrogen and stored at −80°C until use.

### Viability of mPCKS

Viability of mPCKS was determined by measuring ATP content (Roche diagnostics, Mannheim, Germany) and corrected for the total protein content of the sample, which was estimated via the Lowry assay (Bio Rad, Veenendaal, the Netherlands), as described previously (Westra et al., 2013).

### Gene expression

Total RNA was extracted from mPCKS with the RNeasy mini kit (Qiagen, Venlo, the Netherlands) using a Mini-Beadbeater for homogenization, and RNA (1 μg) was reverse transcribed using the Reverse Transcription System (Promega). Subsequently, mRNA expression was evaluated by quantitative real-time polymerase chain reaction (qPCR) performed with a 7900HT qPCR system (Applied Biosystems). Relative fold change was calculated using the 2^−ΔCt^ method using *Gapdh* as reference gene. The TaqMan Gene Expression assays (Thermo Fisher Scientific, Waltham, USA) and SYBR Green primer (Sigma-Aldrich) used are listed in Table 1.

**Table 1:** Primers and antibodies.

Taqman expression assays	Gapdh	Mm99999915_g1
	Il1b	Mm00434228_m1
	Il6	Mm04207460_m1
	Cxcl1	Mm04207460_m1
	Col1a1	Mm00801666_g1
	Fn1	Mm01256744_m1
	Serpinh1	Mm00438058_g1
	Acta2	Mm00725412_s1
SYBR Green primer	Gapdh Pai-1 (Serpine1)	Forward: ACAGTCCATGCCATCACTGC Reverse: GATCCACGACGGACACATTG Forward: GCCAGATTTATCATCAATGACTGGG Reverse: GGAGAGGTGCACATCTTTCTCAAAG
Antibody for Western Blot	Collagen type 1	Primary: Rabbit anti-collagen type 1 (1:2,000; Rockland Immunochemicals, Limerick, USA) Secondary: Goat anti-rabbit HRP (1:2,000, Dako, Santa Clara, USA)
	α-smooth muscle actin (α-SMA)	Mouse anti-α-SMA clone 1A4 (1:5,000; Sigma-Aldrich, St. Louis, USA) Secondary: Rat anti-mouse HRP (1:5,000, Dako)
	Heat-shock protein (HSP47)	Rabbit anti-HSP47 (1:2,000; ab109117 Abcam, Cambridge, USA) Secondary: Goat anti-rabbit HRP (1:2,000, Dako)

### Western blot

Total protein was extracted from mPCKS with ice-cold lysis buffer (30 mM Tris, 150 mM NaCl, 1 mM EDTA, 0.054% Triton X-100, 1 mM Na_3_VO_4_, 10 mM NaF, and 1% SDS) and samples were homogenized (five cycles of 45 s Minibead-beating and 10 min cooling on ice). After 45 min of centrifugation at 16,000 x g, samples were denatured at 75°C for 10 min. A total of 100 μg was separated via sodium dodecyl sulfate–polyacrylamide gel electrophoresis using 10% gels (containing 2,2,2-trichloroethanol, Sigma-Aldrich, for total protein) and blotted onto polyvinylidene fluoride membranes (Trans-Blot Turbo Transfer System, Bio-Rad, Hercules, USA). Membranes were blocked in 5% non-fat milk/TBST (Bio-Rad) and incubated with the primary antibody (Table 1) overnight at 4°C followed by incubation with the corresponding secondary antibody for 1 h. Proteins bands were visualized using the Clarity Western ECL blotting substrate (Bio-Rad) and the ChemiDoc Touch Imaging System (Bio-Rad). Protein expression was normalized using total protein stains for immunoblot.

### Morphology

To assess general morphology, Periodic acid-Schiff (PAS) staining was performed on 2 μm sections from formalin-fixed paraffin-embedded slices. In addition, Picrosirius Red (PSR) staining was used to visualize total collagen levels. Murine PCKS diameter was measured using ImageJ (National Institutes of Health).

### Statistics

Statistics were performed using GraphPad Prism 6.0 via one-way analysis of variance (ANOVA) followed by Dunnett's multiple comparisons test or an unpaired, two-tailed Student's *t*-test as appropriate. The results are expressed as mean ± standard error of the mean (SEM). Regarding the qPCR results, statistics were performed using the ΔCt values, while the data is presented as fold change (2^−ΔCt^). Differences between groups were considered to be statistically significant when *p* < 0.05.

## Results

### Morphology and general viability

**Figure 2:**
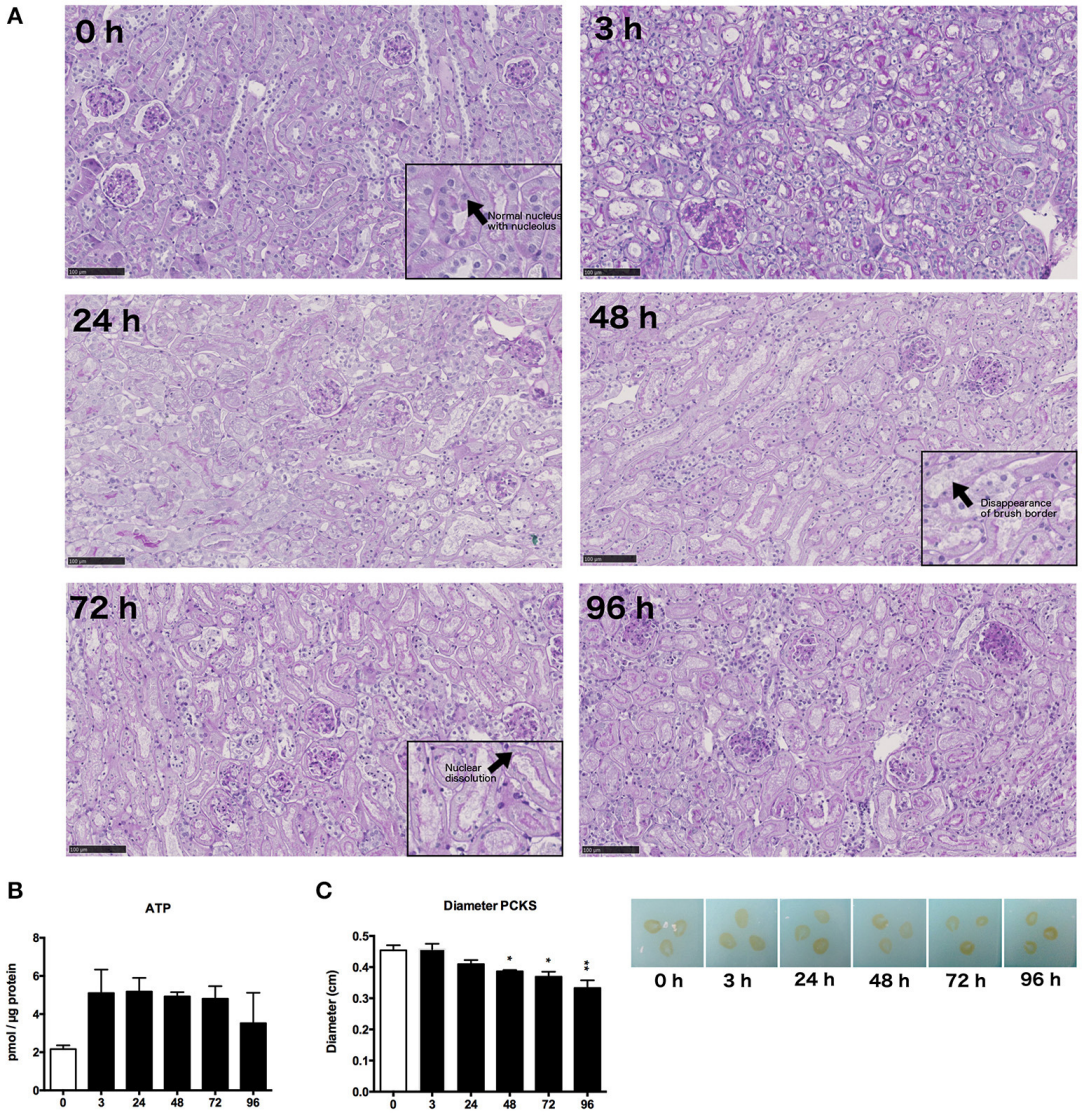
General morphology and viability. **(A)** PAS staining of mPCKS during culture, magnification × 10; insets, magnification × 20, scale bar represents 100 μm. **(B)** Viability measured by ATP content during incubation. **(C)** Slice diameter in cm during incubation. ^*^p < 0.05, ^**^p < 0.001. Data is expressed as mean (±SEM), *n* = 3. PCKS, precision-cut kidney slices.

Morphology of mPCKS was checked with PAS staining at 0, 3, 24, 48, 72, and 96 h of incubation (Figure 2A). As illustrated, kidney slices remained viable up to 48 h of incubation after which the proximal tubular brush border disappeared and most tubular cells became anuclear. Therefore, future experiments were limited to 48 h. Furthermore, the ATP content of the slices increased at the start of culture, after which the levels remained constant (Figure 2B). These results show that mPCKS remained viable for at least 48 h of culturing. Additionally, the diameter of mPCKS reduced during culture (Figure 2C), in accordance with results obtained with human PCKS.

### Early inflammatory and late fibrotic response during incubation

**Figure 3:**
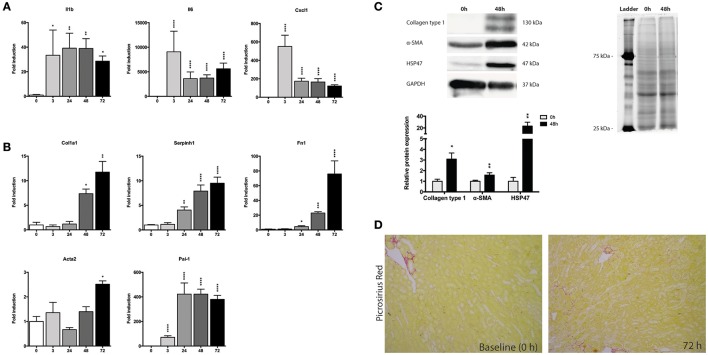
Expression of fibrosis and inflammatory markers during incubation of mPCKS. **(A)** mRNA expression of inflammation markers. **(B)** mRNA expression of fibrosis markers. **(C)** Protein levels of collagen type 1, α-SMA, and HSP47 at baseline and 48 h with representative Western blot images. **(D)** Picrosirius red staining of mPCKS visibly increased at 72 h compared to baseline expression (0 h). ^*^p < 0.05, ^**^p < 0.001, ^***^p < 0.001, ^****^p < 0.0001. Data is expressed as mean (±SEM), *n* = 3–5. Il1b, interleukin 1 beta; Il6, interleukin 6; Cxcl1, C-X-C motif chemokine ligand 1; Col1a1, collagen type I alpha 1; Serpinh1, serine proteinase inhibitor clade H (Heat Shock Protein 47) member 1; Fn1, fibronectin 1; Acta2, alpha 2 smooth muscle actin; Pai-1, Plasminogen activator inhibitor-1; α-SMA (alpha smooth muscle actin); HSP47, heat-shock protein 47; kDa, kilodalton.

Next, we investigated changes in the gene expression of mPCKS during culture, with a focus an inflammation and fibrogenesis. Gene expression of *Il1b, Il6*, and *Cxcl1* markedly increased after 3 h of incubation (Figure 3A). Both *Il6* and *Cxcl1* mRNA levels peaked at 3 h (fold change of 8,715 and 529, respectively), after which the expression levels decreased, although they remained higher as compared to 0 h. The expression pattern of *Il1b* had a nearly constant increase during incubation (118 times fold induction at 48 h) compared to 0 h slices.

We also observed a culture-induced fibrotic response in mPCKS whereby *Fn1* and *Serpinh1* (i.e., heat-shock protein 47, HSP47) are already upregulated after 24 h (respectively 4.5- and 4.0-fold change compared to 0 h), while after 72 h all four markers of fibrosis (*Col1a1, Fn1, Serpinh1*, and *Acta2*) are highly expressed (Figure 3B). Moreover, *Pai-1* gene expression greatly increased at 3 h of incubation (70-fold induction) and even further at 24 h of incubation (420-fold induction) after which levels plateaued. This increase in fibrosis markers was also observed on protein level by western blot (Figure 3C). Collagen type 1 and HSP47 significantly increased at 48 h compared to baseline expression (90 and 98% increase, respectively). These results were further confirmed via a Picrosirius Red staining, which revealed increased collagen levels at 72 h of incubation compared to baseline (Figure 3D).

### Role of TGF-β pathway during incubation of mPCKS

**Figure 4:**
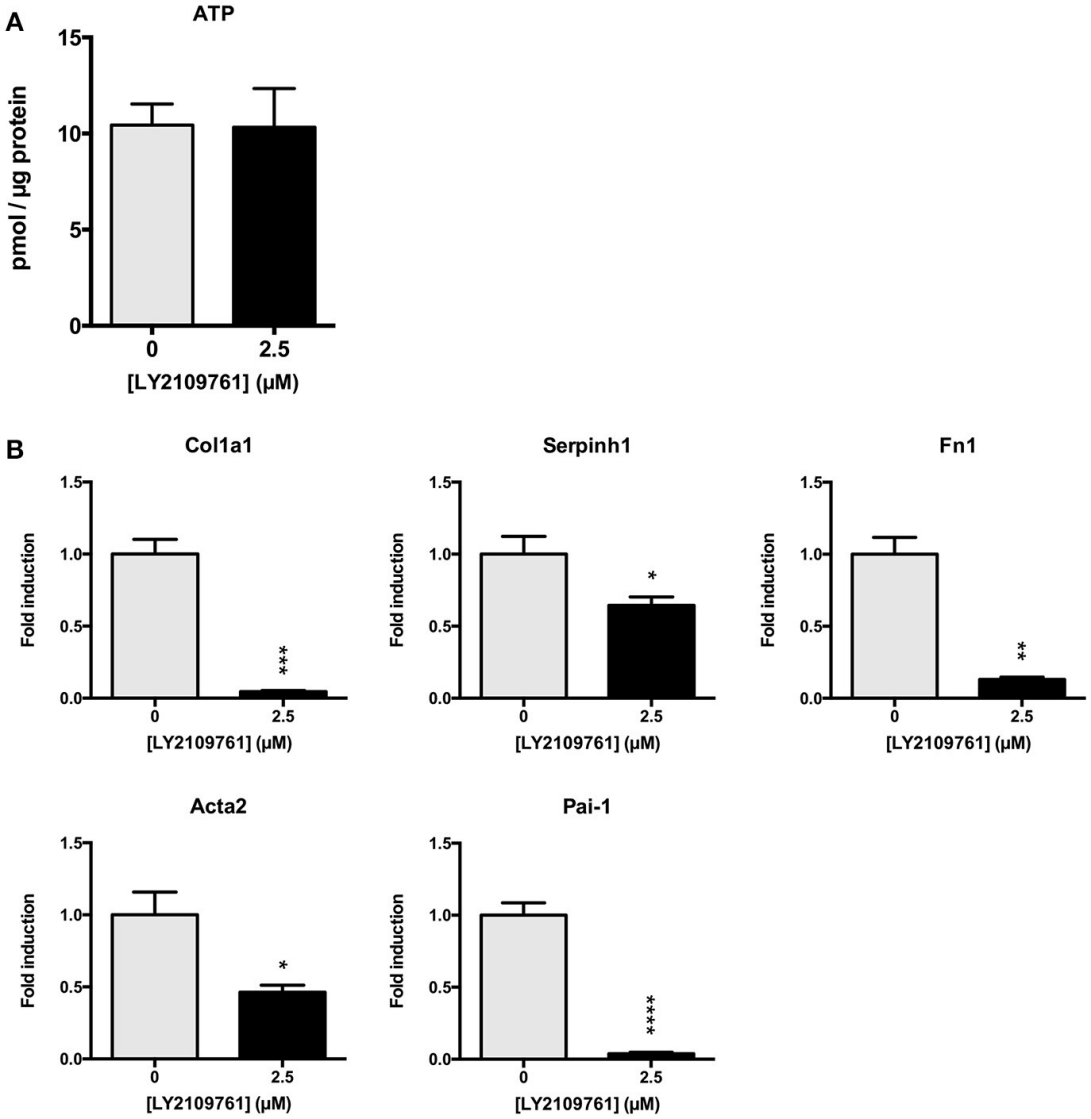
TGF-β receptor inhibition in mPCKS by LY2109761. **(A)** Viability of slices treated with LY2109761 compared to 48 h control. **(B)** Reduction of fibrosis gene expression (Col1a1, Acta2, Serpinh1, Fn1) in slices treated with LY2109761 for 48 h. ^*^p < 0.05, ^**^p < 0.001, ^***^p < 0.001, ^****^p < 0.0001. Data is expressed as mean (±SEM), *n* = 4–5. mPCKS, murine precision-cut kidney slices; Col1a1, collagen type I alpha 1; Serpinh1, serine proteinase inhibitor clade H (Heat Shock Protein 47) member 1; Fn1, fibronectin 1; Acta2, alpha 2 smooth muscle actin; Pai-1, Plasminogen activator inhibitor-1.

**Figure 5:**
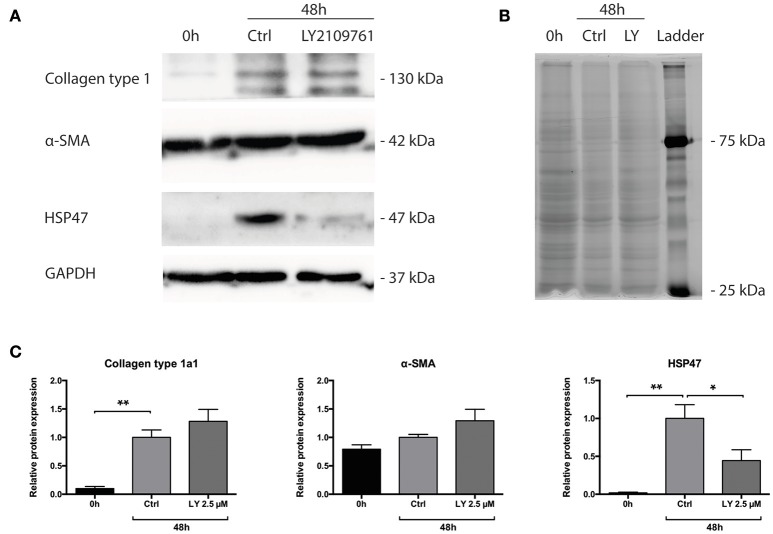
Protein levels of Collagen type 1 alpha 1, HSP47 and α-SMA of PCKS at 0 h, 48 h and treated with LY2109761, a TGF-β receptor type I/II (TβRI/II) dual inhibitor. **(A)** Representative Western blot images of Collagen type 1 alpha 1, HSP47, α-SMA and GAPDH. **(B)** Total protein stain with 2,2,2-trichloroethanol. **(C)** Quantification of detected proteins, after correction for total protein content. ^*^p < 0.05, ^**^p < 0.001. Data is expressed as mean (±SEM), *n* = 4. α-SMA, alpha smooth muscle actin; HSP47, heat-shock protein 47; kDa, kilodalton; LY, LY2109761.

Subsequently, we studied whether the TGF-β pathway is involved in the observed onset of fibrosis in mPCKS. To this end, mPCKS were incubated for 48 h with LY2109761, a selective TGF-β receptor type I/II inhibitor. Our results demonstrated that slice viability, as measured by ATP content, was not affected by LY2109761 (Figure 4A). Furthermore, treatment with LY2109761 significantly reduced the gene expression of all tested fibrosis markers (*Col1a1, Acta2, Serpinh1, Fn1, and Pai-1*) at 48 h, as compared to control (Figure 4B). The inhibitor had the strongest impact on *Col1a1* and *Fn1*, both genes encoding for ECM proteins. Surprisingly, following treatment, *Col1a1* mRNA levels were even lower than in slices at 0 h (1.0 ± 0.1 at 0 h, 6.3 ± 0.6 at 48 h control and 0.3 ± 0.05 at 48 h with LY2109761). In addition, LY2109761 prevented the strong *Pai-1* mRNA induction observed in control slices (96% reduction compared to 48 h control slices). Moreover, we were able to demonstrate a clear decrease in HSP47 protein expression when slices were treated with LY2109761, whilst collagen type 1 and α-SMA expression were not affected (Figures 5A–C).

## Discussion

Translational models of renal fibrosis to develop efficacious antifibrotic therapies are lacking (Stribos et al., 2016a; Klinkhammer et al., 2017) and there is an urgent need to close the gap between *in vivo* studies and clinical trials. PCKS have been around for several decades for pharmacotoxicology studies and recently this *ex vivo* model has been implemented in the fibrosis research field (Poosti et al., 2015; Genovese et al., 2016; Zhang et al., 2016). Strengths of this promising technique are the preserved multicellular heterogeneity and organ architecture, the possibility to perform translational research using human PCKS and the potential to replace/reduce animal experiments.

In the present study, we demonstrate two different applications of mPCKS namely as an *ex vivo* model to study renal fibrogenesis and as a screening tool for antifibrotic therapies such as TGF-β inhibitors. Poosti et al. were the first to use mPCKS to test the efficacy of potential antifibrotic therapies (Poosti et al., 2015). However, they did not fully characterize the ongoing inflammatory/fibrotic processes that take place during mPCKS culture, which is highly important to interpret the results obtained with this model. Our results demonstrated the spontaneous onset of fibrosis during mPCKS culture, as reflected by an increased gene expression of *Col1a1, Serpinh1, Fn1, Acta2, and Pai-1*. Moreover, the acquired fibrotic phenotype was corroborated on a protein level. Additionally, fibrogenesis was preceded by an early inflammatory response on mRNA level, which, although decreasing after 24 h, remained high during culture. This is most probably caused by cell injury due to the slicing procedure as well as a stress response due to incubation at 37°C. Early inflammation, followed by the induction of ECM proteins concurrent with (proximal) tubular atrophy is an important characteristic of renal fibrosis (Farris and Colvin, 2012), which is nicely reflected in mPCKS. Therefore, mPCKS are an ideal model to study renal fibrogenesis. Additionally, preservation of *Acta2* gene and α-SMA protein expression during incubation suggests survival of α-SMA expressing (myo)fibroblasts during culture. In contrast, *Acta2* expression in human PCKS first dramatically decreases after which levels rise again (Stribos et al., 2016b) which might reflect species differences in the fibrotic process. After having extensively characterized the pathological processes in mPCKS, we studied the involvement of the TGF-β pathway herein. To this end, mPCKS were incubated with the TGF-β receptor I/II inhibitor LY2109761 for 48 h. LY2109761 is mostly studied in the context of cancer research, and it has been demonstrated that this inhibitor has antiproliferative effects and increases tumor sensitivity to treatment (Gao et al., 2015; Alsina-Sanchis et al., 2016). Regarding fibrosis, it has been shown to reduce radiation- or bleomycin-induced lung fibrosis (Flechsig et al., 2012; Zhu et al., 2017). Our study confirmed the antifibrotic effect of LY2109761 as we observed a remarkable decrease in the expression of fibrosis genes. Expression of *Pai-1*—a downstream signaling molecule of the TGF-β1 pathway (Krag et al., 2005; Samarakoon et al., 2013)—markedly increased during incubation and was mitigated by LY2109761, indicating that TGF-β1 signaling is one the driving forces for fibrogenesis in mPCKS. Even on a protein level, a significant decrease of HSP47 was observed compared to control. HSP47 expression is closely linked to collagen formation (Razzaque and Taguchi, 1997; Razzaque et al., 1998; Liu et al., 2001; Xiao et al., 2012) and plays an important role in the correct folding and assembly of procollagen molecules (Taguchi and Razzaque, 2007). Additionally, involvement of the protein in fibrotic lesions in the kidney has been established (Abe et al., 2000; Ohba et al., 2005) and when targeted a reduction in renal fibrosis is observed in a UUO animal model (Sunamoto et al., 1998; Xia et al., 2008). Therefore, the observed decrease in HSP47 protein expression is a strong indicator of the antifibrotic efficacy of LY2109761. Importantly, the LY2109761 concentration (2.5 μM) used in this study is low compared to studies in dermal fibroblasts (10–20 μM; Wang et al., 2015, 2016). In conclusion, these studies demonstrate that the TGF-β pathway plays a major role in the onset of fibrosis in mPCKS and indicate that this pathway is an interesting therapeutic target for renal fibrosis.

Limitations of the mPCKS model are the lack of circulating inflammatory and bone-marrow-derived cells contributing to the pathophysiology of renal fibrosis (Lin et al., 2009; Meng et al., 2014), the absence of blood- and urine flow as well as missing interorgan interactions. The latter might be circumvented by co-incubating mPCKS with liver/heart/intestinal slices by using microfluidic biochips (van Midwoud et al., 2010). Additionally, the discrepancy between stable ATP levels, indicating active glycolysis and cellular respiration, and the observed loss of proximal tubular brush borders, reflecting tubular damage, needs to be addressed in future studies to further improve the model.

**Figure 6:**
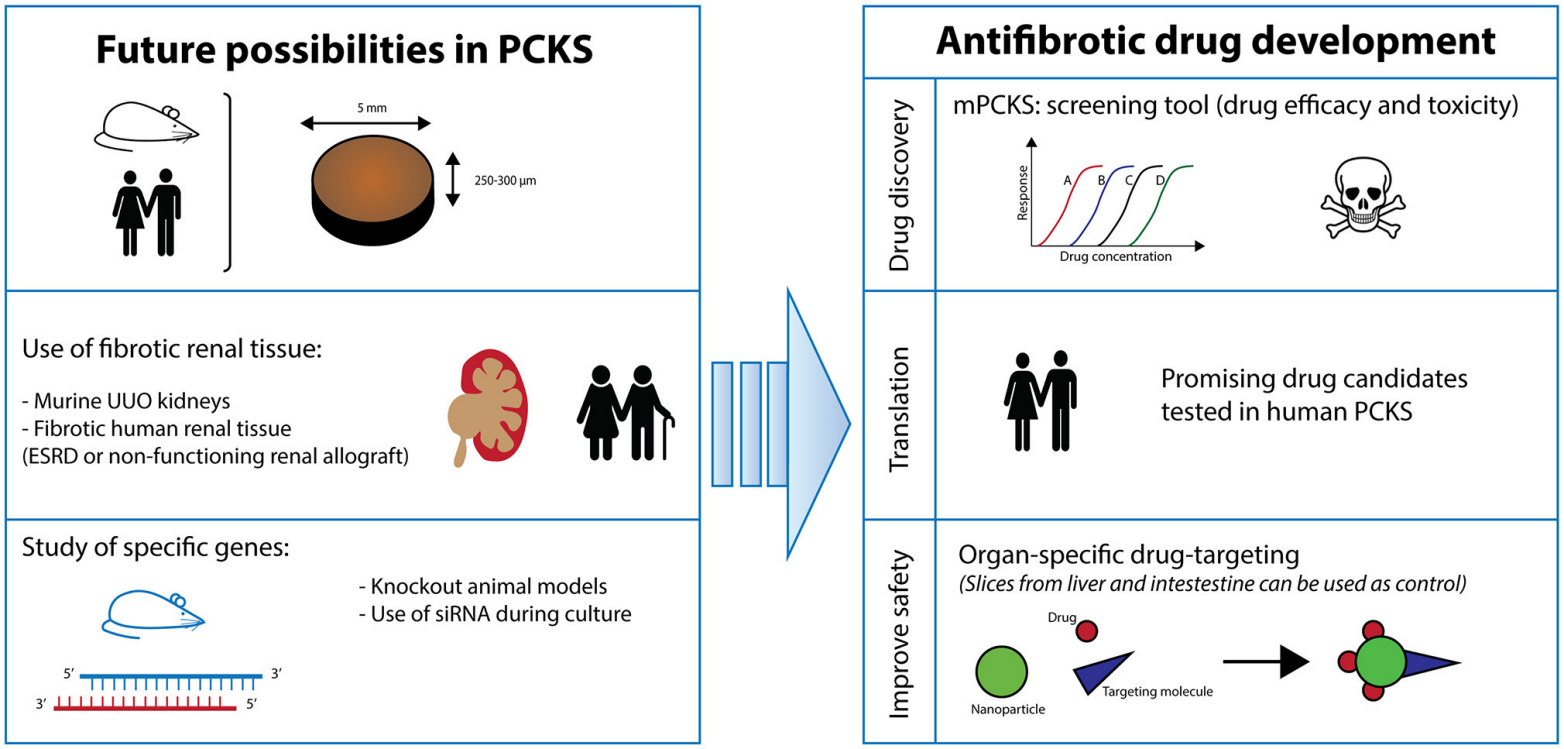
Future perspectives. PCKS, precision-cut kidney slices; ESRD, end-stage renal disease; UUO, unilateral ureteral obstruction.

This *ex vivo* model of PCKS is still in its infancy and offers a vast array of applications (Figure 6). Firstly, one can vary the renal tissue used. Instead of healthy kidneys, diseased renal tissue can be opted for such as from UUO mice. In a similar fashion, the culture of PCKS from human fibrotic tissue from non-functioning renal allografts or ESRD native kidneys is currently being developed in our lab. In the case of mPCKS, specific genes can be studied by using knockout models. Otherwise, siRNA treatment is an alternative option for murine as well as human PCKS, which is currently being studied in our group. Additionally, study of renal disease in PCKS is certainly not limited to fibrosis as this model is also highly suitable to study acute kidney injury.

In conclusion, we present a characterized model of mPCKS to study renal disease (i.e., fibrosis) and show the pivotal role of the TGF-β pathway in the spontaneous onset of fibrosis during incubation. Still, PCKS technology is continuously evolving as a translational model and promises to be an innovative technique that can be used as replacement and reduction for animal experiments.

## Author contributions

ES, MS, HvG, PO, and HM: designed the study; ES: performed the experiments; ES and HM: analyzed the data; MS, HvG, and PO: helped supervise the project; All authors discussed the results and contributed to the final manuscript.

### Conflict of interest statement

The authors declare that the research was conducted in the absence of any commercial or financial relationships that could be construed as a potential conflict of interest.

## Abbreviations

α-SMA: alpha-smooth muscle actin
ANOVA: One-way analysis of variance
CKD: Chronic kidney disease
ECM: Extracellular matrix
ESRD: End-stage renal disease
HSP47: Heat-shock protein 47
mPCKS: Murine precision-cut kidney slices
PAS: Periodic Acid Schiff
PCKS: Precision-cut kidney slices
PSR: Picrosirius Red
qPCR: Quantitative real-time polymerase chain reaction
SEM: Standard error of the mean
siRNA: Small interfering RNA
TGF-β: Transforming-growth factor beta
UW solution: University of Wisconsin cold storage solution.
